# The Prevalence and Associated Risk Factors of Impaired Glucose Regulation in Chinese Adults: A Population-Based Cross-Sectional Study

**DOI:** 10.1155/2015/731583

**Published:** 2015-06-02

**Authors:** Dong Zhao, Nannan Wu, Jing Yang, Simo Liu, Ning Zhang, Xuhong Wang, Haibin Zhang

**Affiliations:** ^1^Beijing Key Laboratory of Diabetes Prevention and Research, 82 South Xinhua Road, Tongzhou District, Beijing 101149, China; ^2^Department of Endocrinology, Beijing Luhe Hospital, Capital Medical University, China; ^3^Department of Cardiology, Beijing Luhe Hospital, Capital Medical University, China

## Abstract

The goal of this study was to determine the prevalence and associated risk factors of impaired glucose regulation (IGR) in the population of Tongzhou, China, and to provide scientific basis for preventive interventions. In the study, the overall age-standardized prevalence of IGR (16.0%) in Tongzhou residents was higher than that in the national population (15.0%). There was no significant geographic difference in prevalence of IGR between urban and rural males. Older age, elevated blood pressure, high serum lipids, overweight, and central obesity were significantly associated with increased risk of IGR.

## 1. Introduction

People with diabetes (DM) are at high risk of heart disease, stroke, and kidney failure [1, 2]. According to the International Diabetes Federation, diabetes caused 4.9 million deaths in 2014 globally [3]. There are 387 million people with diabetes worldwide in 2014, meaning that one in 12 people have diabetes (8.3%). Among them, 77% live in low- or middle-income countries, including China [3]. Diabetes prevalence in Chinese population has increased dramatically over the past 35 years. A cross-sectional national study in 1980 reported that less than 1% of Chinese were diabetic. However, the prevalence of diabetes in Chinese adults rose to 11.6% in 2010, the highest in the world, reported by a study published in 2013. Nearly 113.9 million Chinese adults are suffering from diabetes nowadays. Only one in ten of those have effective glycemic control. In addition, almost half of Chinese adults have prediabetes, according to the study above [4]. Diabetes has become a critical health issue in China. It would not only burden the health care system but also bring societal and financial problems in the future.

Impaired glucose regulation (IGR), including impaired fasting glucose (IFG) and impaired glucose tolerance (IGT), refers to a metabolic state intermediate between normal glucose tolerance and type 2 diabetes [5]. As an important natural history of type 2 diabetes, IGR is suggested by a number of prospective studies to be related to high risk of type 2 diabetes [6, 7]. Compared with epidemiological data on diabetes, data on prevalence of IGR are limited in China. Identifying the epidemic characteristics of IGR could provide an opportunity to intervene in the development of type 2 diabetes and related cardiovascular disease in Chinese population.

As a typical urban fringe district of Beijing, Tongzhou has undergone a dramatic urbanization in the past decade. One result of the transition is the growth of urban residents and the decrease of rural population. To the best of our knowledge, there is only one study that estimated the prevalence of IGR in suburban communities, which was conducted in Guangzhou, Guangdong Province [8]. No study has yet been done to explore the prevalence of IGR in urban fringe of Beijing. Thus, this study aims at accessing the epidemic characteristic of IGR in urban-rural fringe of Beijing.

## 2. Methods

### 2.1. Study Population and Sampling Method

A multistage, stratified, simple random sampling design was used to select participants from 4 neighborhood communities in Tongzhou. The communities were chosen to represent the variety of economic development and geographical distribution. A total of 1105 subjects aged 18–40 years were recruited in the study. Data of 1069 participants were successfully collected. The overall response rate was 96.74%.

### 2.2. Data Collection

A questionnaire was designed according to the Method of Chinese Chronic Disease Surveillance (2010) and administrated by trained interviewers. Written informed consents/oral consents were obtained from all participants. The questionnaire included information on social-demographic characteristics (age, sex, occupation, education level, etc.), manual labor, family history of diabetes, medical history, and diet habits. Physical examination was also done and height, weight, waist and hip circumference, and blood pressure were measured using a standard protocol. WHR was calculated through dividing waist circumference by hip circumference. BMI was calculated via dividing weight in kilograms by height in meters squared.

For all participants, blood samples were collected to determine FPG, FINS, TC, TG, high-density lipoprotein cholesterol (HDL-C), and low-density lipoprotein cholesterol (LDL-C) after an overnight fast of at least 8 hours. Participants without known diabetes were given an oral glucose tolerance test (OGTT) to measure the 2-hour plasma glucose (2hPG). They were given a standard 75-g glucose solution to drink and other blood samples were collected 2 hours later. The blood samples were collected using vacuum tubes containing sodium fluoride and were sent to local laboratories immediately. Qualities of onsite data collection and laboratory test were stringently controlled to ensure the validity of the data.

### 2.3. Diagnostic Criteria

According to the World Health Organization (WHO) 1999 criteria [5], diabetes was defined as (1) FPG level of 7.0 mmol/L or higher or (2) 2hPG level of 11.1 mmol/L or higher. IFG was defined as (1) FPG level between 6.1 mmol/L and 7.0 mmol/L and (2) 2hPG level lower than 7.8 mmol/L. IGT was defined as (1) FPG level lower than 7.0 mmol/L and (2) 2hPG level between 7.8 mmol/L and 11.1 mmol/L. “Normal” was defined as (1) no history of diabetes, (2) FPG level lower than 6.1 mmol/L, and (3) 2hPG level lower than 7.8 mmol/L. Hypertension was defined by WHO 1999 guideline as (1) systolic blood pressure (SBP) of 140 mmHg or higher or (2) diastolic blood pressure (DBP) of 90 mmHg or higher [6]. Dyslipidemia was defined as (1) TC level of 5.18 mmol/L or higher, (2) TG level of 1.70 mmol/L or higher, (3) HDL-C level of 1.04 mmol/L or lower, or (4) LDL-C level of 3.37 or higher, according to 2007 Guidelines for the Management of Dyslipidemia in Chinese Adults. Overweight was defined as BMI between 24 Kg/m^2^ and 28 Kg/m^2^, and obesity was defined as BMI of 28 Kg/m^2^ and higher, according to a study that focused on optimal cutoff points of BMI for Chinese adults [9]. Central obesity was defined as waist circumference of 85 cm or larger in males and 80 cm or larger in females [9].

### 2.4. Statistical Analysis

All the continuous variables were presented as mean ± standard deviation and categorical variables as percentage. Age-adjusted prevalence was estimated from age-specific prevalence and the national age distribution in 2010 census using standardization method. The differences in proportions were examined using the chi-square test. A multiple unconditional logistic regression model was applied to explore the association between the potential risk factors and IGR, as well as the odds ratios adjusted for other risk factors. Two-sided *P* value was used with the significance level of 0.05. All of the analyses were performed with SPSS Statistics 17.0 for Windows (SPSS Inc.).

## 3. Results

Of the 1069 study subjects, 323 (30.2%) were male and 746 (69.8%) were female. A total of 51.5% (551) participants were urban residents, and 48.5% (518) were rural residents. The sex difference in IGR prevalence is not significant (*P* > 0.05). By and large, the circumstance, TG, blood pressure, and FPG increased with age in both men and women regardless of residence. There was no clear trend for HDL-C in terms of age. Several diagnostic measures are presented in Table 1.

Table 2 showed the age-specific and age-standardized prevalences of diabetes and IGR by sex and residence. The prevalence of diabetes was higher in urban than in rural residents, regardless of gender (20.5% in urban and 14.9% in rural men, 14.6% in urban and 12.3% in rural women). Similarly, the prevalence of IGR in urban men was above the rate in rural men (19.0% in urban and 18.2% in rural men). The difference was not significant (*P* > 0.05). However, the prevalence of IGR in women showed an opposite trend (13.9% in urban and 19.0% in rural women). IGR rate was significantly higher in rural than in urban women (*P* < 0.05). Furthermore, the prevalence of IGR was higher in urban population than in rural residents for both men and women after age standardization.

Generally, IGR prevalence increased with age in both men and women except that it decreased after hitting a peak of 25.0% at the age of 50 to 59 years in rural men and after reaching a maximum of 29.2% at the age between 60 and 69 years in urban women. The difference of IGR rates across age groups was significant for both males and females (*P* < 0.05). For both men and women, IGR prevalence was significantly higher among those over 50 years compared with the rest (*P* < 0.05).

IGR was slightly more prevalent in men than in women (18.6% in men and 16.2% in women) without significant difference (*P* > 0.05). This conclusion held after age standardization (16.4% in men and 16.1% in women).

Age, blood pressure, TG, TC, BMI, and waist circumstance were significantly positively associated with IGR after adjusting for multiple risk factors (*P* < 0.05). People with older age, elevated blood pressure, increased serum lipids, overweight, obesity, or central obesity were at high risk of IGR. We did not find any significant association between IGR and manual labor and family history of diabetes (*P* > 0.05) (Table 3). However, family history of diabetes was a significant risk factor of IGR for women (*P* < 0.05).

Through the multivariate logistic model, we estimated that IGR risk in overweight and obese people was 1.4 times higher than that in normal-weight population. Central obesity and hypertension increased the risk of IGR by 1.7 and 1.5 times, respectively. Every one-mmol/L-increase of TG and TC corresponded to 1.9- and 1.3-time increase in the risk of IGR, respectively (Table 3).

## 4. Discussion

This cross-sectional study demonstrated an IGR prevalence of 16.9% in Tongzhou population. IGR was slightly more prevalent in men than in women, both before and after age standardization. In addition, IGR prevalence increased with age in both men and women regardless of residence. The prevalence of IGR turned out to be significantly higher over 50 years in men, which could be explained by the increasing blood pressure, serum lipids, and some other risk factors of IGR in this population. Similarly, the increase of IGR prevalence was dramatic after 60 years in women, consistent with a previous study [10]. As shown in a number of studies, the decline of estrogenic hormones after menopause could affect the *β*-cell function, insulin resistance, and glucose metabolism, which in turn contributed together to the rise of IGR prevalence in menopausal women [1, 11–13]. Therefore, improvement in blood sugar monitoring in elderly population may play an important role in IGR prevention as well as early diagnosis of diabetes.

Another main issue explored in this study was the residential difference in IGR in a typical urban-rural fringe. The crude prevalence of IGR was significantly higher in rural women compared to urban women. But such significant residential difference was not seen in men. After age standardization, IGR prevalence in urban residents was shown to be higher than that in rural population, for both men and women. However, the difference was not significant. The rapid economic growth and urbanization of rural area in Tongzhou may explain a similar prevalence of IGR in urban and rural population. On the one hand, the lifestyle of rural population has changed. Intake of high-fat and high-calorie food has increased while physical activities have decreased in rural residents. On the other hand, the gap between urban and rural areas on access and quality of medical system exacerbates the elevation of IGR rate among rural population. This finding suggests that preventive intervention of diabetes should be applied regardless of economic development, especially in urban fringe district where the border between urban and rural areas has been blurring.

Furthermore, we found that age, blood pressure, TG, TC, BMI, and waist circumstance were significantly positively associated with IGR after adjusting for multiple risk factors, in agreement with previous studies [14–16]. People with older age, high blood pressure, elevated serum lipids, overweight, obesity, or central obesity were at increased risk of IGR. Controlling of blood pressure, serum lipids, obesity, and central obesity is the key for preventing diabetes and related complications.

In this study, we used the 1999 WHO criteria. We also noticed that the cutoff of IFG was set up as 5.6 mmol/L instead of 6.1 mmol/L in the 2003 ADA criteria. Given its better sensitivity in predicting future diabetes, it can be reasonably expected that the prevalence of IGR and diabetes will be even higher than the numbers presented in our study.

The strength of this study was that it was the first population-based study to determine the epidemic characteristic of IGR in urban fringe district of Beijing. In addition, this study conducted a comprehensive exploration on more key risk indicators of diabetes compared to similar studies in china.

The limitation of this study was the imbalanced gender among our sample population, which may be explained by the time and site of subject selection. Since sex difference in IGR may exist, the overall prevalence of IGR and diabetes in this study may be different from the true prevalence in Tongzhou.

## Figures and Tables

**Table 1:** General characteristics of study subjects.

Age (years)	Mean ± SD					
	Waist circumstance (cm)	TG (mmol/L)	SBP (mmHg)	DBP (mmHg)	FPG (mmol/L)	HDL (mmol/L)
20–29						
Male						
Urban	83.94 ± 9.86	1.49 ± 1.93	114.46 ± 12.80	73.24 ± 7.94	4.91 ± 0.76	1.07 ± 0.24
Rural	79.38 ± 11.35	0.86 ± 0.34	112.54 ± 10.80	72.83 ± 9.22	5.33 ± 0.31	1.15 ± 0.164
Female						
Urban	72.47 ± 11.40	0.81 ± 0.46	105.78 ± 12.20	69.55 ± 8.09	4.83 ± 0.43	1.32 ± 0.29
Rural	75.23 ± 11.41	0.67 ± 0.28	110.59 ± 9.10	71.54 ± 7.18	4.99 ± 0.39	1.38 ± 0.24
30–39						
Male						
Urban	90.06 ± 11.18	2.96 ± 4.2	120.71 ± 18.17	78.93 ± 13.03	5.31 ± 1.05	2.58 ± 1.20
Rural	90.89 ± 11.37	1.75 ± 1.28	122.21 ± 16.78	80.91 ± 12.43	5.74 ± 1.21	1.12 ± 0.25
Female						
Urban	76.62 ± 9.50	0.92 ± 0.08	108.03 ± 11.98	72.17 ± 9.70	5.14 ± 0.65	2.19 ± 0.48
Rural	79.23 ± 9.18	1.01 ± 0.73	113.84 ± 15.98	75.17 ± 11.86	5.36 ± 0.70	1.31 ± 0.31
40–49						
Male						
Urban	90.21 ± 9.14	2.89 ± 3.67	120.15 ± 15.39	81.87 ± 11.5	6.36 ± 2.07	0.99 ± 0.33
Rural	90.30 ± 9.67	1.36 ± 0.99	133.92 ± 17.63	84.35 ± 10.49	5.95 ± 1.05	1.22 ± 0.32
Female						
Urban	81.19 ± 10.2	1.28 ± 0.94	120.08 ± 17.30	79.44 ± 10.70	5.61 ± 1.88	1.28 ± 0.30
Rural	85.57 ± 9.51	1.18 ± 1.24	125.56 ± 18.12	82.83 ± 11.82	5.59 ± 1.66	1.27 ± 0.31
50–59						
Male						
Urban	91.75 ± 12.10	1.58 ± 0.71	123.39 ± 10.54	81.43 ± 9.20	5.80 ± 0.97	1.08 ± 0.22
Rural	91.69 ± 10.10	1.24 ± 0.34	137.35 ± 17.18	87.82 ± 10.92	6.16 ± 2.04	1.24 ± 0.34
Female						
Urban	84.12 ± 9.34	1.52 ± 1.12	126.42 ± 18.80	79.91 ± 10.20	5.70 ± 1.74	1.26 ± 0.28
Rural	88.22 ± 9.40	1.27 ± 0.28	131.55 ± 18.06	84.18 ± 10.01	5.86 ± 1.23	1.27 ± 0.28
60–69						
Male						
Urban	85.91 ± 6.32	1.10 ± 0.47	130 ± 13.54	80.23 ± 7.15	6.46 ± 1.42	1.20 ± 0.28
Rural	90.24 ± 7.97	1.28 ± 0.66	140.84 ± 18.36	87.88 ± 12.87	5.65 ± 1.08	1.33 ± 0.33
Female						
Urban	97.23 ± 5.41	1.56 ± 0.90	132.49 ± 15.18	86.85 ± 47.73	6.79 ± 2.77	1.28 ± 0.35
Rural	91.63 ± 12.05	2.29 ± 2.41	138.79 ± 15.18	83.57 ± 11.34	6.46 ± 2.12	1.14 ± 0.30
70–74						
Male						
Urban	91.35 ± 10.90	1.20 ± 0.71	144.12 ± 23.68	80.29 ± 11.52	6.76 ± 2.08	1.17 ± 0.33
Rural	96.66 ± 9.66	1.08 ± 0.38	141.11 ± 21.03	81.11 ± 10.24	5.30 ± 0.63	1.27 ± 0.15
Female						
Urban	90.39 ± 10.97	1.59 ± 0.71	131.62 ± 17.40	76.52 ± 8.18	6.37 ± 1.65	1.38 ± 0.29
Rural	91.60 ± 8.32	1.41 ± 0.49	142.00 ± 10.95	93.00 ± 14.83	5.48 ± 0.38	1.37 ± 0.22

**Table 2:** Age-specific and age-standardized prevalence of diabetes and impaired glucose regulation in study subjects, by sex and residence.

Variable	Age- (years) specific prevalence *N* (%)							Age- (years) standardized prevalence %
	20–29	30–39	40–49	50–59	60–69	70–74	Total	20–74
DM								
Male								
Urban	1 (3.2)	2 (14.3)	10 (29.4)	7 (25.0)	5 (22.7)	5 (29.4)	30 (20.5)	22.1
Rural	0 (0)	4 (11.1)	9 (18.0)	7 (14.6)	4 (10.5)	3 (33.3)	27 (14.9)	10.3
Total	1 (3.2)	6 (12.0)	19 (22.6)	14 (18.4)	9 (15.0)	8 (30.8)	57 (17.4)	14.4
Female								
Urban	0 (0)	2 (3.3)	11 (11.7)	15 (14.7)	13 (27.1)	7 (30.4)	48 (14.6)	14.3
Rural	0 (0)	5 (5.5)	10 (8.8)	17 (17.3)	9 (25.7)	1 (20.0)	42 (12.3)	9.4
Total	0 (0)	7 (4.6)	21 (10.1)	32 (16.0)	22 (26.5)	8 (28.6)	90 (13.4)	11.9
IGR								
Male								
Urban	2 (6.3)	1 (7.1)	4 (11.8)	7 (25.0)	6 (27.3)	8 (47.1)	28 (19.0)	18.4
Rural	0 (0)	6 (16.7)	4 (8.0)	12 (25.0)	8 (24.2)	2 (22.2)	32 (18.2)	14.0
Total	2 (6.3)	7 (14.0)	8 (9.5)	19 (25.0)	14 (25.5)	10 (38.5)	60 (18.6)	16.4
Female								
Urban	5 (6.6)	7 (11.5)	10 (10.6)	15 (14.7)	14 (29.2)	5 (21.7)	56 (13.9)	15.8
Rural	0 (0)	5 (5.5)	21 (18.6)	26 (26.5)	11 (31.4)	2 (40.0)	65 (19.0)	14.0
Total	5 (6.6)	12 (7.9)	31 (15.0)	41 (20.5)	25 (30.1)	7 (25.0)	121 (16.2)	16.1

**Table 3:** Adjusted OR and 95% CI of IGR by associated risk factors.

Variable	*β*	*P*	OR	95% CI
Age	0.262	0.000	2.307	1.713–2.411
BMI	0.372	0.000	1.447	1.357–1.511
Waist circumstance	0.441	0.019	1.703	1.700–1.782
TG	0.661	0.000	1.933	1.899–1.980
TC	0.277	0.015	1.333	1.577–2.315
Hypertension	0.413	0.017	1.516	1.412–1.693
Heavy manual labor	0.222	0.510	0.071	0.692–1.020
Family history of DM	0.011	0.600	0.780	0.531–1.021
